# Identification of the nasopharyngeal carriage of *Neisseria meningitidis* by 16S rRNA Gene sequencing in asymptomatic adolescents and young adults in Cartagena, Colombia (2019–2020).

**DOI:** 10.1016/j.bjid.2022.102330

**Published:** 2022-02-15

**Authors:** Marlon Macias-Mendoza, Alfredo Montes-Robledo, Cindy Arteta-Acosta, Rosa Baldiris-Avila, Wilfrido Coronell-Rodríguez

**Affiliations:** a Microbiologia Clínica y Ambiental Research Group, University of Cartagena, Cartagena, Colombia; b Faculty of Medicine, University of Cartagena, Cartagena, Colombia; c Professor, University of Sinu, Cartagena, Colombia; d Doctorate in Medical Science, Faculty of Medicine, University of Chile, Santiago, Chile; e Intensive Care and Obstetric Research Group (GRICIO). University of Cartagena, Cartagena, Colombia

**Keywords:** *Neisseria meningitis*, Carriage, Serogroups, Nasopharynx

## Abstract

The bacterium *Neisseria meningitidis*, a strictly human pathogen, can cause meningitis, meningococcemia, sepsis, and death; repeatedly it scause outbreaks around the world. The frequency of asymptomatic carriage is often high in adolescents and young adults, increasing the invasive meningococcal disease risk and likelihood of transmission. However, detailed analyses of meningococcal carriage in this population in Colombia, particularly in coastal areas, are lacking. In this study, the prevalence and characteristics of *Neisseria meningitidis* carriage were evaluated in asymptomatic adolescents and young adults (11-25 years old) in Cartagena, Colombia. Oropharynx samples were collected from participants between August and December 2019. The phenotypic identification of bacteria was performed by conventional methods and biochemical testing. Molecular identification to the species level was performed by 16S rRNA gene sequencing. In total, 12 of 648 samples were positive for *Neisseria meningitidis* by 16S rRNA sequencing, indicating a prevalence of 1.9%. Isolates were classified into four invasive serogroups (A, B, C, and W) by a comparative sequence analysis of the ribosomal gene. Despite the occurrence of meningococcal disease in Cartagena city in the last several years, the frequency of oropharyngeal carriage in adolescents and young adults was low. Serogroup A had not been previously reported in nasopharyngeal samples in Colombia. This is the first report of *Neisseria meningitidis* on the Colombian Caribbean coast based on 16S rRNA sequencing and is expected to guide the development of vaccination and follow-up strategies.

## Introduction

*Neisseria meningitidis* (Nm) is a Gram-negative bacterium that causes invasive meningococcal disease (IMD). The IMD has a characteristically rapid course, in which fatalities often occur within 24 hours, reach up to 50-80% of untreated cases; even with optimal treatment, 10-15% of patients die.^1^ Up to 30% of survivors develop long-term sequelae.^2^

According to the capsular polysaccharide composition, Nm is currently classified into 12 serogroups; however, only six (i.e., A, B, C, Y, W, and X) are responsible for the majority of IMD worldwide.^3^ These bacteria colonize the nasopharynx, this asymptomatic carriage is age-related, with the highest peak during adolescence with rates of up to 23.7-50%.^4^ In Colombia, the prevalence of meningococcal carriage in adolescent has been reported in 6.8%.^5^ Humans are the unique reservoir of this pathogen.^4^^,^^6^ Transmission occurs by the spread of respiratory drops during casual contact, rather than directly from patients with IMD.^7^ Because most transmissions occur between carriers, effective preventive strategies, including meningococcal conjugate vaccines, are important to decrease carriage rates.

Meningococcus is characterized by high diversity, with epidemic strains belonging to different serogroups.^1^ In Colombia, IMD occurs as sporadic cases with occasional outbreaks, and the surveillance system is based on the mandatory notification of IMD cases as per the standardized case definition. The notification of cases of bacterial meningitis in Colombia is carried through the National Public Health Surveillance System "SIVIGILA" which provides the country's data to the System of Surveillance Networks of Agents Responsible for Pneumonias and Bacterial Meningitis "SIREVA". The incidence has remained between 0.13-0.22/100,000 inhabitants between 2013 and 2019.^8^, ^9^, ^10^ IMD is mostly caused by *N. meningitidis* serogroup B (MenB) and C (MenC), responsible for outbreaks occurring in Cartagena city, Colombia between 2012 and 2016.^9^^,^^11^ In the present study, analyses of the prevalence of carriage, phenotypic properties, and species-level identification were performed by 16S rRNA gene sequencing of asymptomatic adolescents and young adults in Cartagena.

## Methods

### Study design

A cross-sectional study of adolescents and young adults was performed between August and December 2019. Students aged 11–25 years were selected by stratified sampling, considering the stratification of student by geographical area of schools, university, and grade of school. In Cartagena, the educational establishments are geographically distributed in five administrative units (The Country, Virgin and Touristic, Santa Rita, Industrial and bay, and Rural) with public and private schools: 72 (16.6%), 97 (22.4%), 71 (16.4%), 147 (34.0%), and 45 (10.4%), respectively; and from official page of Cartagena Department of Education (http://www.sedcartagena.gov.co/) we had access to the list of schools. The sample size was calculated by stratified random sampling based on published data; for a carriage prevalence of 6.8%^5^ with a 2.5% error and 95% confidence level, the required sample size was 388. Considering a 10% loss rate, the minimum number of samples required was 427.

Several secondary schools and Universities in the different areas were selected randomly maintaining the proportion of geographical area. Permission from each institution to develop the project was requested and, finally, those that gave their approval were included. Inside the institutions, in each grade, we selected randomly the participants based on assistance list, maintaining the stratification.

Participants and parents/guardians (for subjects younger than 18 years) were informed of the study aim and procedures in writing. All participants or parents/guardians provided written informed consent. Before sample collection, the students answered a structured questionnaire with items on personal information; age; socioeconomic status (in Colombia there are six class levels defined by income, housing, and environmental characteristics, where more economically precarious conditions are classified as status one and the wealthiest as status six)^12^, we classified it into three groups (low 1 and 2; medium 3 and 4; high 5 and 6); siblings numbers; number of people in house and per room, smokers at home; attending parties; to belong to a teenage group; number of people kissed in the last two weeks; sexual activities and number of sexual partners in the last three months; and consumption of alcohol, tobacco and illegal drugs. Trained staff collected oropharyngeal samples from the posterior wall of the oropharynx.

### Isolation, culture, and identification of bacterial strains

Samples were obtained with a sterile calcium alginate swab from the posterior wall of the oropharynx, stored in Stuart agar, and transported, as soon as possible, to the Clinical and Environmental Microbiology laboratory at the University of Cartagena. The same day, samples were seeded in modified Thayer-Martin culture medium and incubated at 37° C with 5% CO_2_ and 50% humidity. All cultures were examined at 24, 48, 72, and 96 hours. The positive culture plates were subcultured in chocolate agar. The genus was preliminarily identified by conventional methods (i.e., Gram stain, oxidase test, and biochemical characterization) and using an automated identification system (MicroScan). All colonies were preserved on brain heart infusion (BHI) broth and glycerol at -80° C.

### Purification and biochemical identification

Species identification was based on colony morphology on chocolate agar, oxidase tests, and sugar fermentation. Taxa were confirmed using a panel of biochemical tests (MicroScan). All colonies were preserved in BHI and glycerol at -80° C.

### DNA isolation

Presumptive *Neisseria* colonies were cultivated on chocolate agar for 24 hours at 37° C. Ten colonies were taken for genomic DNA extraction using a commercial kit (DNeasy Blood & Tissue Kit; Qiagen, Hilden, Germany) following the manufacturer's instructions. The quality and concentration of all DNA samples were evaluated by UV-VIS spectrophotometry (NanoDrop 2000; Thermo Scientific, Waltham, MA, USA).

### Determination of the 16S rRNA gene sequence

The 16 rRNA gene, considered as a molecular signature to identify members of bacterial communities, is conserved among prokaryotes with specific variable regions.

The 16S rRNA gene was amplified by PCR using universal primers 27F (5′-AGAGTTT GATCMTGGCTCAG-3′) and 1492R (5′-GGTTACCTTGTTACGACTT-3′).^13^ PCRs were performed in a final volume of 50 µL, containing 2 µL of DNA (50 ng/µL), 2 µL of 1 × buffer, 3 mM MgCl_2_, 0.2 mM each dNTP, 1 U Taq Polymerase (Fermentas, Waltham, MA, USA), 0.4 μM direct and reverse primers, and deionized water. PCR amplification was performed using a thermocycler (Mastercycler Nexus-Eppendorf) as follows: denaturation at 95° C for five minutes, followed by 30 cycles of denaturation at 94° C for 45 seconds, alignment at 56° C for 45 seconds, and extension at 72° C for 90 seconds, and a final extension of 72° C for 10 minutes. Products were separated on 1.5% agarose gels in 1 × TBE buffer stained with ethidium bromide (0.5 mg/mL) and visualized under UV light. PCR products were purified using the QIAquick PCR Purification Kit (Qiagen), according to the manufacturer's instructions. The purified PCR products were sequenced (ABI PRISM 3500 Automatic Sequencer) using the vector pGEM®-3Z (Promega, Madison, WI, USA) as a positive control.

### Analysis of the 16S rRNA gene sequence

Sequence chromatograms were edited using Chromas (version 2.6.4; Technelysium, South Brisbane, Australia). The 16S rRNA gene sequences were assembled using the free trial version of SeqMan Pro 13 (DNASTAR, Madison, WI, USA). The homology of the 16S rRNA gene sequences was verified with 16S rRNA sequences from other organisms in the GenBank database using the BlastN algorithm (http://www.ncbi.nih.gov/BLAST/). Then, inconsistencies or errors were manually deleted using EditSeq. For identification, BLAST searches against the NCBI database were performed (https://blast.ncbi.nlm.nih.gov/Blast.cgi?PROGRAM=blastn&PAGE_TYPE=BlastSearch&LINK_LOC=blasthome). New sequences were verified, uploaded to the database, and assigned codes.

### Construction of phylogenetic trees

Phylogenetic trees were built using the neighbor-joining method and Kimura 2-parameter model, with 1,000 replicates to evaluate support for internal branches.^14^ Nucleotide similarity was evaluated using MEGA X.^15^ *Neisseria gonorrhoeae* strain NCTC 8375 (NR_026079) was used as an outgroup as control. As proposed by Rosselló & Amann,^16^ <95%, 95–97.5%, and >97.5% were the thresholds for taxonomic assignments at the family, genus and species levels, respectively. The phylogenetic trees were built with different serogroups of Nm in the GenBank database with the following accession codes: AF310588, AF310582, and AF310581 (serogroup A-MenA); AY132121, AY132143, and AY12119 (MenB); AF310346, AF382291, and AY132095 (C); AF310551, AF310607, and AF310605 (serogroup W-MenW); AF337915, AF337923, and AF310516 (serogroup Y-MenY).

### Ethics approval

The Ethics Committee of University of North, Colombia approved the present study (October 29, 2015, Code N°. 134).

### Statistical analysis

Medians with interquartile range (IQR) for quantitative variables and proportions for qualitative variables as well as frequencies (%) of carriage were calculated. Univariate and logistic regression analyses were performed to identify risk factors for meningococcal carriage, *p-*values (e.g., p < 0.05 indicated a significant difference), odd ratios (OR), and 95% confidence intervals (95% CI) were calculated using IBM SPSS statistics 22.

## Results

### Characteristics of teenagers

Twenty institutions were contacted in four months, 16 responded, seven declined to participate, and nine agreed to participate in the project. In total, 649 samples were obtained. One sample was excluded owing to handling errors, leaving 648 samples, including 245 from high school students and 403 from university students.

Table 1 summarizes the demographic characteristic of the subjects. The median age of participants was 18 years [IQR 15 - 20] and 65.1% were female. In total, 56% of students belonged to a low socioeconomic level. Ninety-two percent of adolescents had siblings (median, two siblings), and almost 70% of siblings were younger 16 years old. The students lived in households with a median of four individuals and two individuals per room, with a maximum of six individuals sharing the same room. Twelve adolescents (1.8%) were oropharyngeal carriers of *Neisseria* at the time of the study.

**Table 1:** Sociodemographic characteristics data of 648 adolescents and distribution by carrier status, August – December 2019, Cartagena – Colombia.

*Univariate associations*						*Logistic regression analysis*	
Variable		TotalN 648 (%)	CarriersN 12 (%)	Non-carriersN 636 (%)	*P*-value	*OR*	*95% CI*
Total sample	*High School*	245 (37.8)	7 (58.3)	238 (37.4)	0.13		
	*University*	403 (62.1)	5 (41.7)	398 (62.6)			
Sex	*Female*	422 (65.1)	7 (58.3)	414 (65.1)	0.62		
Age, years old	*Median [IQR]*	18 [15 - 20]	15 [13–18.5]	18 [15–20]	-		
	*11 – 15*	184 (28.4)	7 (58.3)	177 (27.8)	0.02	3.51	1.08 -11.32
	*16 – 25*	464 (71.6)	5 (41.6)	459 (72.1)	-		
Socioeconomic status	*Low*	365 (56.3)	7 (57.3)	358 (56.2)	0.98		
	*Middle*	230 (35.4)	4 (33.3)	226 (35.5)			
	*High*	53 (8.1)	1 (8.3)	52 (8.1)			
Siblings*	*Median [IQR]*	2 [3;5]	2.0 [1–3.5]	2.0 [1– 3]	0.71		
	*<10 years*	203 (31.3)	4 (33.3)	199 (31.3)	0.88		
	*11-15 years*	227 (35.0)	3 (25.0)	224 (35.2)	0.46		
	*16-21*	275 (42.4)	6 (50.0)	269 (42.3)	0.59		
	*≥22*	209 (32.3)	4 (33.3)	205 (32.2)	0.93		
N° people in house	*Median [IQR]*	4 [4 - 5]	4.5 [4–5.5]	4 [4–5]	0.28		
N° people per room	*Median [IQR]*	2 [1 -2]	2 [1.5–2.0]	2 [ 1–2]	0.25		
Smokers at home	*Yes*	74 (11.4)	1 (8.3)	73 (11.5)	0.73		
Parties	*No*	151 (23.3)	1 (8.3)	150 (23.6)	0.46		
	*Biannual*	100 (15.4)	3 (25)	97 (15.3)			
	*Once/month*	177 (27.3)	2 (16.7)	175 (27.5)			
	*2-3 times/month*	146 (22.5)	5 (41.7)	141 (22.2)			
	*Once a week*	64 (9.9)	1 (8.3)	63 (9.9)			
	*No response*	10 (1.5)	0 (0)	10 (1.6)			
To belong to a teenage group	*Yes*	233 (36.0)	3 (25)	230 (36.2)	0.65		
Kiss in the last 2 weeks	*Yes*	367 (56.6)	7 (58.3)	360 (56,6)	0.90	0.54	0.09 – 3.03
Kiss more than one person	*Yes*	52 (8.0)	4 (33.3)	48 (7.5)	0.001	5.88	1.68 – 20.53
Sexual partner	*Yes*	260 (40.1)	5 (41.7)	255 (40.1)	0.95	2.82	0.58 – 13.69
N° of sexual partners in the last 3 months	*Not answered*	25 (3.9)	0 (0.0)	25 (3.9)	0.92		
	*1*	215 (33.2)	5 (41.7)	210 (33)			
	*2*	26 (4.0)	0 (0.0)	26 (4.1)			
	*3*	5 (0.8)	0 (0.0)	5 (0.8)			
	*4*	2 (0.3)	0 (0.0)	2 (0.3)			
Alcohol	*Yes*	248 (38.3)	5 (41.7)	243 (38.2)	0.93		
Smoking	*Yes*	26 (4.0)	0 (0)	26 (4.1)	0.72		
Illegal drugs (marijuana and cocaine)	*Yes*	10 (1.5)	0 (0)	10 (1.6)	0.88		

⁎ Adolescents may have several siblings with different ages.

### Risk factors for pharyngeal carriage

Table 1 provides a comparison of risk factors surveyed between carriers and non-carriers, regarding social behavior, 56% of adolescents reported having kissed in the last two weeks but comparing the frequency of kissing, we found a significant difference (33% versus 7.5%, *p* 0.001), 16.7% of carriers had kissed three or more people versus 3.1% in non-carriers. In logistic regression analysis, students under 16 years of age and kissing more than one person in the last two weeks increased the probability of being a carrier of *N. meningitidis* OR 3.51 (95%CI 1.08-11.32), and OR 5.88 (95% CI 1.68 – 20.53) respectively*.* There was no significant difference in having a sexual partner and their frequency, consuming alcohol, smoking, and using illegal drugs.

### Microbiology and molecular biology

#### Conventional technique

**Fig. 1:**
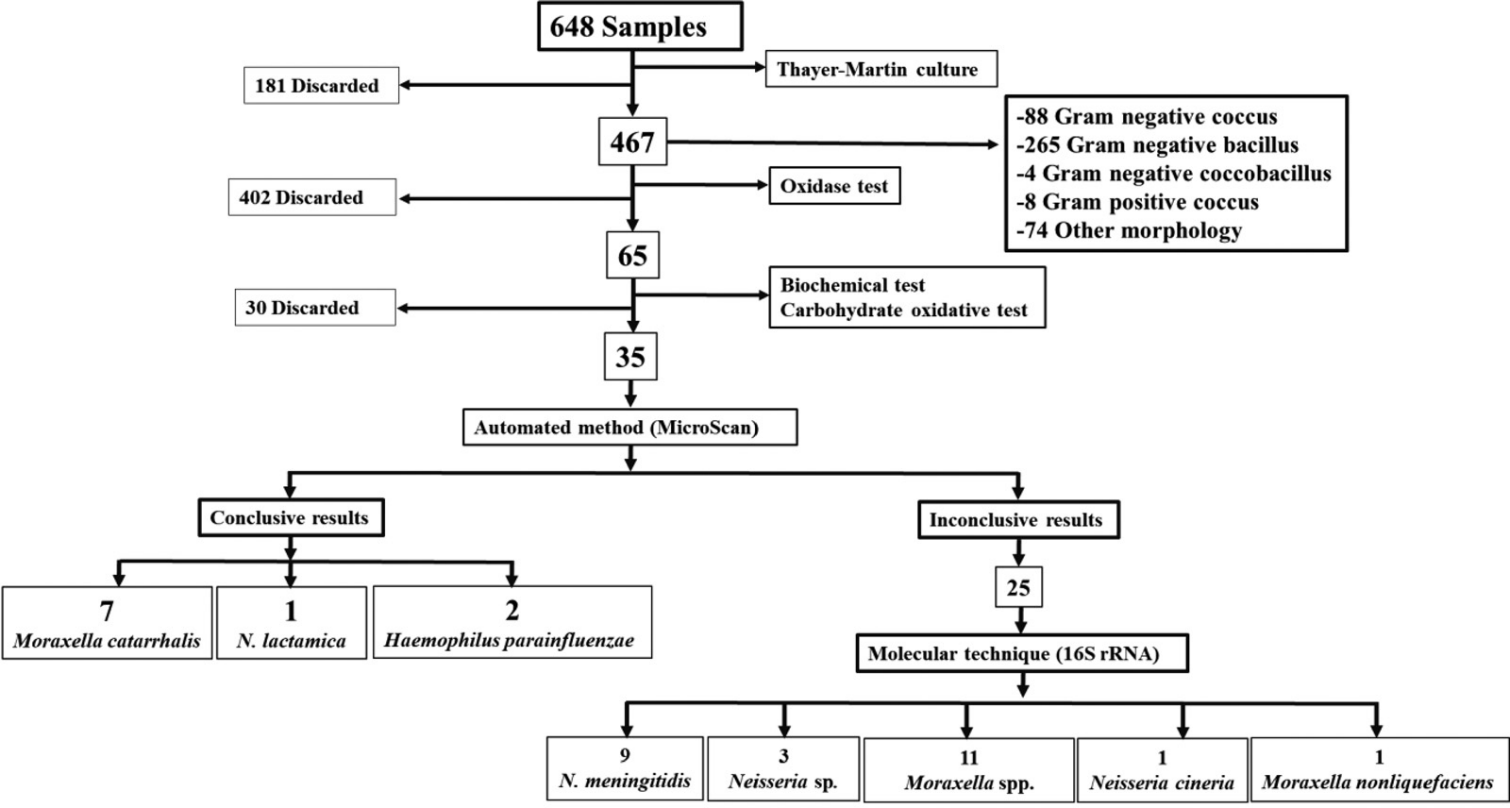
Processing and identification of strains, isolated from adolescents by conventional, automated, and molecular methods in Cartagena, Colombia 2019.

By conventional microbiological techniques and the automated MicroScan method, we identified 35 strains, 10 of this had conclusive results (7 *Moraxella catarrhalis*, 2 *Haemophilus parainfluenzae* and 1 *Neisseria lactamica*), which will be used in future studies. The remaining 25 isolated had inconclusive results by MicroScan, because the machine requested visual confirmation from the analyst for the sugar fermentation panel, therefore, they underwent molecular testing. Fig. 1.

#### Molecular technique

Molecular identification based on 16S rRNA sequencing of the 25 strains yielded the following results: 11 (44%) *Moraxella* spp., 1 (4%) *Moraxella nonliquefaciens*, 1 (4%) *Neisseria cineria*, 9 (36%) *Neisseria meningitidis*, and 3 (12%) *Neisseria* sp. (Fig. 1).

The 12 isolates categorized as *N. meningitidis* (9 strains) and *Neisseria* sp. (3 strains) were verified and submitted to the GenBank database (access codes for each strain MT279986, MT279987, MT279988, MT279989, MT279990, MT279991, MT279992, MT279993, MT279994, MT279995, MT279996 and MT279997).

**Fig. 2:**
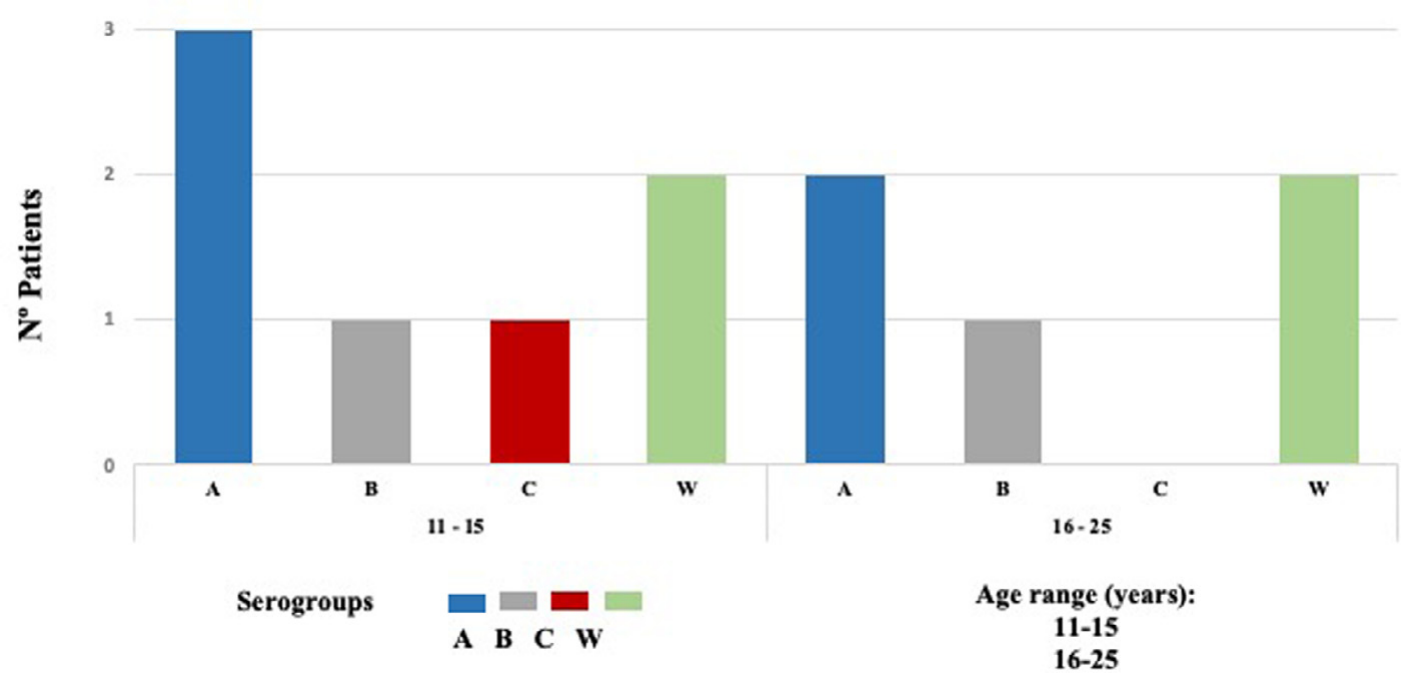
Distribution of 12 Neisseria meningitidis isolated in adolescents by age-ranged, Cartagena-Colombia 2019.

Nm was isolated in 12 of 648 samples, indicating a carriage prevalence of 1.9%. Twelve isolated strains were subclassified into four invasive serogroups (MenA, MenB, MenC, and MenW). Based on sequence homology of the 16S ribosomal gene determined by a BLAST analysis, the bacterial strains were assigned to serogroups as follows: five serogroup MenA (42%), two MenB (17%), one MenC (8%), and four MenW (33%) (Fig. 2).

**Fig. 3:**
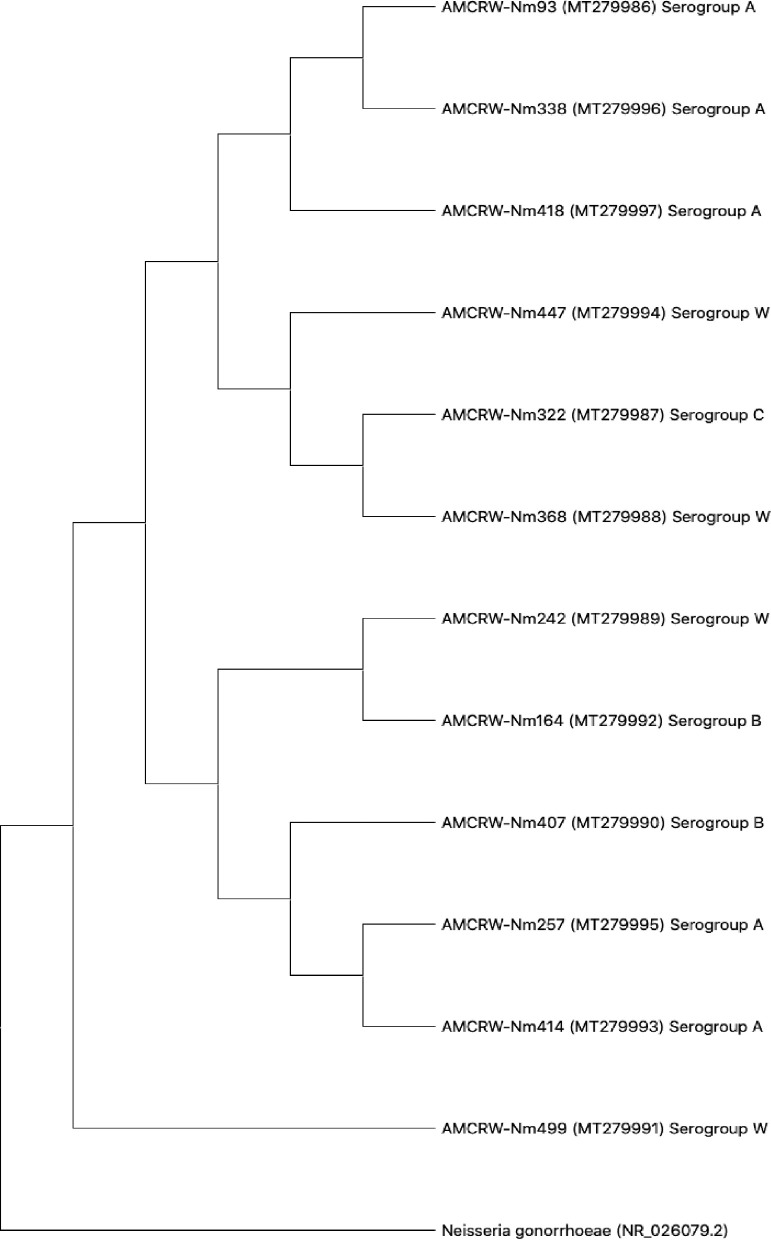
Phylogenetic relationships among Neisseria meningitidis isolates from adolescents in Cartagena, Colombia 2019.

Three strains showed slightly lower nucleotide sequence similarities (88–93%) in comparisons using BLAST; a phylogenetic tree was generated for each isolate (Supplementary material Fig. S1 - S12). Furthermore, we generated a phylogenetic tree to evaluate the overall relationships among the resulting clades. As shown in Fig. 3, 12 isolates corresponding to Nm showed similarity values of ≥97%, and the observed differences did not support the separation of clades. These results suggested that the strains in each clade corresponded to isolates within a serogroup.

## Discussion

We observed a nasopharyngeal carriage prevalence of 1.9%, which was lower than previous estimates in adolescents of carriage studies in Bogota-Colombia (6.8%), and also lower than in other countries of south America like Chile, Argentina and, Brazil (6.5%, 9.4%, and 9.9%, respectively).^5^^,^^17^, ^18^, ^19^

The dynamics of nasopharyngeal carriage of Nm is complex, with multiple factors such as environmental, host and bacterial converge. It has been described a relationship between a higher frequency of meningococcal infections and climate. Regions where the climate is dry with low relative humidity and a higher number of circulating dust particles report an increase in meningococcal infections.^20^ In consequence a possible explanation for the lower prevalence of carriage in Cartagena's adolescents could be the climatic and geographic difference between Bogotá and Cartagena. Bogotá is located at 2,600 meters above sea level, with dry climate, low relative humidity and low temperature; unlike Cartagena has a higher relative humidity, rainy and tropical climate.^21^ It is also possible that the low prevalence of nasopharyngeal carriage between outbreak periods (every 7-10 years), a low endemicity in the country, or transitory carriage could explain our results, which is difficult to demonstrate due the methodology of the present study.^22^^,^^23^ Nevertheless, our estimate was similar to the reported prevalence in Mexico (1.9% in children and 2.9% in adolescents)^24^ and Venezuela (1.5% in a sample of schoolchildren).^23^ Accordingly, it may be necessary to analyze other age groups, time periods, and cities to better understand the dynamics of oropharyngeal carriage.

Multiple risk factors have been identified for nasopharyngeal carriage and IMD, such as overcrowding, low socioeconomic status, sharing bedrooms, community housing, active and passive smokers, attending bars or discos, kissing, viral infections, working as microbiologists, and military members.^4^^,^^25^ In our study, adolescents aged 11-15 years had seven out of 12 Nm isolated, and the logistic regression showed that they had three times (95% CI 1.08-11.32) more risk to have carriage of *N. meningitidis*. This contrasts to data published by MenAfriCar where the highest prevalence of nasopharyngeal carriage of 4.9% was reported in 5-14 years versus 3.6% in 15-29 years group.^26^ Likewise, we found that adolescents kissing more than one person had five times (95% CI 1.68–20.53) more risk to be carrier. Interestingly, Moreno et al. found association with practice of oral sex OR 1.70 (95% CI 1.02–2.75).^5^

Colombia does not include conjugate vaccines against meningococcus in its expanded immunization plan. Instead, they are only used for outbreak control, because the low incidence rate of IMD (0.2 cases per 100,000 inhabitants) in Colombia.^27^ Nonetheless, we consider that Ministry of Health should include the vaccination, at least in high-risk populations, as the World Health Organization recommends for countries with an incidence <2 cases per inhabitants by year, but a complete benefit will be the full vaccination regimen in infants and adolescents to remove the carriage status.^5^^,^^19^^,^^23^^,^^24^^,^^28^^,^^29^

The association between carriage and the incidence of IMD remains controversial. Some studies have found that a high rate of carriage is related to a higher incidence of disease, whereas others have found the opposite relationship.^30^^,^^31^ Meningococcus can show temporary or transitory colonization of the nasopharynx and can trigger invasive disease or be transmitted to another individual, which may trigger the disease.^32^^,^^33^ We identified *Neisseria lactamica, Neisseria cinerea, Moraxella nonliquefaciens*, and *Moraxella catarrhalis*. These bacteria have been described as commensal upper respiratory tract bacteria. *N. lactamica*, in particular, could develop immunity against Nm, functioning as a suppressor of this pathogen.^34^

Our use of microbiological and molecular biology methods increased the detection rate of Nm and, consequently, enabled accurate estimates of the prevalence of nasopharyngeal carriage.^35^ Sequence variation in the 16S rRNA gene suggests that it could contribute to the identification of different clones of Nm circulating in a specific area.^36^

Furthermore, conventional phenotype-based techniques are limited with respect to the differentiation and identification of species in the genus *Neisseria*. Accordingly, a phylogenic analysis was performed based on 16S rRNA sequences. However, this approach is limited by a low resolution, capsule switching, or the high gene transfer rate between bacterial isolates. Results should be confirmed with other molecular techniques, such as multilocus sequence typing.^37^ Whole genome sequencing has recently been used as a higher precision technique.^38^

In Cartagena, reported outbreaks have been attributed only to serogroup B^10^^,^^22^; however, circulating serogroups may vary over time. In this study, we detected serogroups other than B (i.e., A, C, and W) in nasopharyngeal samples, and these could be responsible for future meningococcal infections in the city.

The incidence of IMD in the United States has fluctuated over time. In Latin America, the first outbreaks caused by MenA occurred in Brazil (1945 and 1971), and in Chile (1978). Since these, no outbreaks of MenA have been reported on the American continent, the last report was in Colombia in 1989^23^^,^^39^; MenB and MenC are the most prevalent types in Europe, North America, and Australia, but MenW has had an increase in the last two decades, especially in the United Kingdom and the southern cone of Latin America including Argentina and Chile.^40^, ^41^, ^42^ Besides the increases of cases, there was an increased mortality rate (from 10% to 30%).^43^ Since we found MenA in our study, it should be considered a potential cause of IMD in the region.

In Colombia, MenC is currently the most prevalent type in almost all areas, accounting for most outbreaks. However, on the Atlantic coast and especially in Cartagena (where this study was conducted), from 2010 to date, outbreaks have been caused by MenB.^5^^,^^11^^,^^22^^,^^44^ The oropharyngeal carriage of meningococcus in Cartagena city has not been evaluated previously, and future cases of meningococcal disease may involve serogroups other than B. Therefore, our results are expected to contribute to the planning and development of prevention programs.^45^

IMD is a dynamic, unpredictable pathology with extensive geographic and temporal variation in serogroups.^46^ As mentioned previously, although MenA was the first to cause outbreaks or epidemics in the Americas, it has disappeared from the continent. However, it has remained the most prevalent serogroup in Africa, especially in the meningitis belt in the sub-Saharan region, where is responsible for almost all cases of IMD, and also in Asia.^23^^,^^47^ Changes in serogroup frequencies may correspond to public health interventions, such as vaccines or antibiotics, in addition to environmental factors, human behavior, mass events (e.g., Jamboree and Hajj pilgrimage) and globalization.^48^, ^49^, ^50^, ^51^ With respect to globalization, immigration in Colombia increased from 2017 to 2019 by 252%, 2,499,936 individuals in 2017 to 6,758,800 in 2019, including immigrants from Africa and Asia.^52^ On the Colombian Atlantic Coast, there is a settlement of immigrants in transit to other countries. This pattern provides a potential explanation for the detection of MenA in our carriage study. However, whole genome sequence analyses of the strains identified by the 16S sequencing technique are needed to distinguish between the appearance of a new circulating serogroup or changes in the clonal complex (cc) attributed to the capsular switch of Nm.

In a phylogenetic analysis, we observed that MenW is associated with MenC and MenB (Fig. 3). This relationship can be attributed to the gene composition of this serogroup, as reported by the United States Centers for Disease Control and Prevention (CDC).^53^ MenW can show similar behavior to that of MenC and even MenB due to the shared cc ET37/ST11; however, there are few studies of isolates in Cartagena to characterize these genetic features of Nm strains.^53^ It should be noted that the sequence analysis showed that there is a direct relationship between the internal nodes that connect all the branches, evidencing that MenW is associated with each of the branches; however, one clade only contains strains belonging to MenA. This observed characteristic can be attributed to the fact that the strains found and associated to MenW do not present a relationship within the cc with the strains of this study; therefore, the clades that connect the branches of the strains belonging to MenA are interconnected with each other, without associating the other serogroups found in this study.

Otherwise, to create the grouping and make the distribution of N. meningitidis, we had a limitation: few reports of 16S gene sequences in Nm isolates in the databases consulted were found, and the strains and serogroups sequences classified as “non-groupable” by researchers were not available. To explore the sequences of this study, we constructed a phylogenetic tree, analyzed considering the proximity according to the maximum similarity of our genome sequences with those found in the different databases, for which reason non-grouping strains were found in this study. It is recommended a whole-genome sequence for future work (development research).

Another limitation of this study was the cross-sectional methodology; therefore, transient carriage was not detected. Alternatively, we could have overestimated the prevalence of pharyngeal carriage (6.8%) based on estimates for other cities in Colombia,^5^ leading to the underestimation of the required sample size and consequently a lack of statistical power to identify risk factors among carriers. Finally, this study was performed in a single city in Colombia, and the results may not be representative of the true carriage rates in the whole country.

Despite these limitations, this is the first study of meningococcal carriage in teenagers from a Caribbean city in Colombia, and geography may be a major determinant of carrier status. Furthermore, this is the first study of meningococcus in Cartagena to use molecular biology techniques, i.e., 16S sequence analyses, for validation; this easily accessible, inexpensive, and fast tool could provide important information in places where complete genomic sequencing cannot be obtained. Importantly, using this technique, we detected MenA, which has not been previously reported in Colombia. More broadly, our results, and particularly the detection of serogroup A, have important implications for public health and may guide the development of strategies for the prevention of meningococcal disease.

## Conflicts of interest

The authors declare no conflicts of interest.

## References

[1] Borrow R, Alarcón P, Carlos J (2017). The global meningococcal initiative: global epidemiology, the impact of vaccines on meningococcal disease and the importance of herd protection. Expert Rev Vaccines.

[2] Wang B, Clarke M, Thomas N, Howell S, Afzali HHA, Marshall H. (2014). The clinical burden and predictors of sequelae following invasive meningococcal disease in Australian children. Pediatr Infect Dis J.

[3] Zimmer SM, Stephens DS. (2004). Meningococcal conjugate vaccines. Expert Opin Pharmacother.

[4] Christensen H, May M, Bowen L, Hickman M, Trotter CL. (2010). Meningococcal carriage by age: a systematic review and meta-analysis. Lancet Infect Dis.

[5] Moreno J, Hidalgo M, Duarte C, Sanabria O, Gabastou JM, Ibarz-Pavon AB. (2015). Characterization of carriage isolates of neisseria meningitidis in the adolescents and young adults population of Bogota (Colombia). PLoS ONE [Internet].

[6] Caugant DA, Hoiby EA, Magnus P (1994). Asymptomatic carriage of Neisseria meningitidis in a randomly sampled population. J Clin Microbiol.

[7] Cooper LV, Kristiansen PA, Christensen H, Karachaliou A, Trotter CL. (2019). Meningococcal carriage by age in the African meningitis belt: a systematic review and meta-analysis. Epidemiol Infect [Internet].

[8] Safadi MAP, Berezin EN, Arlant LHF. (2014). Meningococcal disease: epidemiology and early effects of immunization programs. J Pediatr Infect Dis Soc [Internet].

[9] INS (2020).

[10] Velez-van Meerbekea A, Medina Silva N, Besada Lombana S, Mojica Madero JA. (2017). Epidemiología de la enfermedad por meningococo en Colombia. Infectio [Internet].

[11] Moreno J, Sanabria O, Saavedra SY, Rodríguez K, Duarte C. (2015). Phenotypic and genotypic characterization of Neisseria meningitidis serogroup B isolates from Cartagena, Colombia, 2012-2014. Biomedica.

[12] Mina L. (2004). Estratificación socioeconómica como instrumento de focalización. Economía y desarrollo [Internet].

[13] Cowan ST, Steel KJ, Barrow GI, Feltham RKA (1993). Cowan and Steel's manual for the identification of medical bacteria.

[14] Kumar S, Stecher G, Li M, Knyaz C, Tamura K (2018). MEGA X: molecular evolutionary genetics analysis across computing platforms. Mol Biol Evol [Internet].

[15] Stecher G, Tamura K, Kumar S. (2020). Molecular Evolutionary Genetics Analysis (MEGA) for macOS. Mol Biol Evol [Internet]..

[16] Rosselló-Mora R. (2001). The species concept for prokaryotes. FEMS Microbiol Rev [Internet].

[17] Díaz J, Cárcamo M, Seoane M (2016). Prevalence of meningococcal carriage in children and adolescents aged 10–19 years in Chile in 2013. J Infect Public Health [Internet].

[18] Gentile A, Della Latta MP, Bloch M (2021). https://dx.plos.org/10.1371/journal.pone.0247991.

[19] Cassio de Moraes J, Kemp B, de Lemos APS (2015). Prevalence, risk factors and molecular characteristics of meningococcal carriage among Brazilian adolescents. Pediatr Infect Dis J.

[20] López EL, Debbag R. (2012). Enfermedad meningocóccica: siempre presente. Cambios en los serogrupos en el Cono Sur. Rev Chil Infectol [Internet].

[21] IDEAM (2021). http://www.ideam.gov.co/documents/21021/418894/Caracter%C3%ADsticas+de+Ciudades+Principales+y+Municipios+Tur%C3%ADsticos.pdf/c3ca90c8-1072-434a-a235-91baee8c73fc.

[22] Boletín epidemiológico semanal, INS (2019). Meningitis bacteriana y enfermedad meningocóccica. Semana Epidemiol.

[23] Safadi MA, Gonzalez-Ayala S, Jakel A, Wieffer H, Moreno C, Vyse A. (2013). The epidemiology of meningococcal disease in Latin America 1945-2010: an unpredictable and changing landscape. Epidemiol Infect.

[24] Espinosa de los Monteros LE, Aguilar-Ituarte F, Jiménez-Rojas LV, Kuri P, Rodríguez-Suárez RS, Gómez-Barreto D. (2009). Prevalence of Neisseria meningitidis carriers in children under five years of age and teenagers in certain populations of Mexico City. Salud pública Méx [Internet].

[25] Rosenstein NE, Perkins BA, Stephens DS (2001). Meningococcal disease. N Engl J Med [Internet].

[26] Trotter CL, Greenwood BM. (2007). Meningococcal carriage in the African meningitis belt. Lancet Infect Dis.

[27] Vespa Presa J, Abalos MG, Sini de Almeida R, Cane A. (2019). Epidemiological burden of meningococcal disease in Latin America: A systematic literature review. Int J Infect Dis [Internet]..

[28] WHO (2011). Meningococcal vaccines: WHO position paper, November 2011. Wkly Epidemiol Rec.

[29] Rodriguez P, Alvarez I, Torres MT (2014). Meningococcal carriage prevalence in university students, 18-24 years of age in Santiago, Chile. Vaccine [Internet]..

[30] Olsen SF, Djurhuus B, Rasmussen K (1991). Pharyngeal carriage of Neisseria meningitidis and Neisseria lactamica in households with infants within areas with high and low incidences of meningococcal disease. Epidemiol Infect.

[31] Fernández S, Arreaza L, Santiago I (1999). Carriage of a new epidemic strain of Neisseria meningitidis and its relationship with the incidence of meningococcal disease in Galicia, Spain. Epidemiol Infect.

[32] Quagliarello V. (2011). Dissemination of *Neisseria meningitidis*. New Eng J Med [Internet].

[33] Carbonnelle E, Hill DJ, Morand P (2009). Meningococcal interactions with the host. Vaccine [Internet].

[34] Deasy AM, Guccione E, Dale AP (2015). Nasal Inoculation of the Commensal Neisseria lactamica Inhibits carriage of neisseria meningitidis by young adults: a controlled human infection study. Clinic Infect Dis.

[35] Pace D, Pollard AJ. (2012). Meningococcal disease: clinical presentation and sequelae. Vaccine [Internet]..

[36] Sacchi CT, Whitney AM, Reeves MW, Mayer LW, Popovic T. (2002). Sequence diversity of Neisseria meningitidis 16S rRNA genes and use of 16S rRNA gene sequencing as a molecular subtyping tool. J Clin Microbiol.

[37] Diallo K, MacLennan J, Harrison OB (2019). Genomic characterization of novel Neisseria species. Sci Rep [Internet].

[38] Marjuki H, Topaz N, Rodriguez-Rivera LD (2019). Whole-genome sequencing for characterization of capsule locus and prediction of serogroup of invasive meningococcal isolates. Mellmann A, editor. J Clin Microbiol [Internet].

[39] INS. Vigilancia por laboratorio de Nm 1987-2019 [Internet]. Disponible en: http://www.ins.gov.co/buscador-eventos/Paginas/Info-Evento.aspx

[40] Valenzuela MT, Mañalich J, Díaz J (2019). Plan de acción nacional frente a la emergencia de la cepa W-135 responsable de enfermedad meningocócica invasora en Chile, 2012-2013. Rev Méd Chile [Internet]..

[41] Instituto de Salud Pública de Chile (2020).

[42] Villena R, Valenzuela MT, Bastías M, Santolaya ME. (2019). Meningococcal invasive disease by serogroup W and use of ACWY conjugate vaccines as control strategy in Chile. Vaccine [Internet].

[43] Olea A, Matute I, González C (2017). Case−control study of risk factors for meningococcal disease in Chile. Emerg Infect Dis [Internet].

[44] Coronell-Rodríguez W, Arteta-Acosta C. (2018). 1985. Atypical symptoms and high mortality associated with Serogroup B meningococcal disease in Cartagena Colombia 2012–2016. Open Forum Infectious Diseases [Internet].

[45] Maiden MCJ, Frosch M. (2012). Can we, should we, eradicate the meningococcus?. Vaccine.

[46] Wilhelm B J, Villena M.R. (2012). Historia y epidemiología del meningococo. Rev chil pediatr [Internet].

[47] LaForce FM, Konde K, Viviani S, Préziosi M-P. (2007). The meningitis vaccine project. Vaccine [Internet].

[48] Badahdah A-M, Rashid H, Khatami A, Booy R. (2018). Meningococcal disease burden and transmission in crowded settings and mass gatherings other than Hajj/Umrah: a systematic review. Vaccine [Internet].

[49] Muttalif AR, Presa JV, Haridy H, Gamil A, Serra LC, Cané A. (2019). Incidence and prevention of invasive meningococcal disease in global mass gathering events. Infect Dis Ther [Internet].

[50] Yezli S. (2018). The threat of meningococcal disease during the Hajj and Umrah mass gatherings: a comprehensive review. Travel Medicine and Infectious Disease [Internet].

[51] Lucidarme J, Hill DMC, Bratcher HB (2015). Genomic resolution of an aggressive, widespread, diverse and expanding meningococcal serogroup B, C and W lineage. J Infect [Internet].

[52] Wabgou M, Vargas D, Carabalí JA. (2012). Las migraciones internacionales en Colombia. Investig desarro [Internet].

[53] Mayer LW, Reeves MW, Al-Hamdan N (2002). Outbreak of W135 Meningococcal Disease in 2000: Not emergence of a new W135 strain but clonal expansion within the electophoretic Type–37 complex. J INFECT DIS [Internet].

